# Potential of acetaminophen on the sublingual microcirculation and peripheral tissue perfusion of febrile septic patients: prospective observational study

**DOI:** 10.1186/s13613-024-01251-z

**Published:** 2024-02-10

**Authors:** R. Domizi, E. Damiani, A. Carsetti, L. Graciotti, A. D. Procopio, C. Scorcella, E. Casarotta, P. Giaccaglia, A. Donati, E. Adrario

**Affiliations:** 1 Anesthesia and Intensive Care Unit, Azienda Ospedaliera Universitaria Delle Marche, Via Conca 71, 60126 Ancona, Italy; 2 Department of Biomedical Sciences and Public Health, Università Politecnica Delle Marche, Via Tronto 10/a, 60020 Ancona, Italy; 3 Department of Clinical and Molecular Sciences, Università Politecnica Delle Marche, Ancona, Italy; 4 Clinic of Laboratory and Precision Medicine, IRCCS INRCA, Ancona, Italy

**Keywords:** Sepsis, Septic shock, Acetaminophen, Microcirculation, Tissue perfusion, Hemodynamic

## Abstract

**Background:**

Acetaminophen (ACT) has been studied in septic patients with detectable plasmatic levels of cell-free hemoglobin (Hb), where it demonstrated to inhibit the hemoprotein-mediated lipid peroxidation and oxidative injury, with a potential of beneficial effect on the endothelium. On the basis of this background, the aim of this study was to evaluate the sublingual microcirculation and the peripheral tissue perfusion before-and-after administration of ACT on clinical judgment in a cohort of febrile septic and septic shock patients.

**Methods:**

Prospective observational study. 50 adult septic and septic shocks treated with ACT for pyrexia, where the sublingual microcirculation and the peripheral tissue perfusion with Near Infrared Spectroscopy (NIRS) and vascular occlusion test (VOT) were evaluated before ACT (t0), after 30 min (t1) and after 2 h (t2). Cell-free Hb and the markers of oxidative stress and endothelial damage were measured at t0 and t2.

**Results:**

The study showed a significant increase of the density of the perfused small and total vessels of the sublingual microcirculation 30 min after the infusion of ACT; it also showed an increase of the Microvascular Flow Index (MFI) and a decrease in the heterogeneity of the flow.

At a peripheral muscular level, we found an acceleration in the reperfusion curve after VOT at t1, expression of a higher reactivity of the microvasculature.

**Conclusions:**

ACT infusion did not show a clear correlation with cell-free Hb; however, it exhibited protective effect toward the microcirculation that was evident in particular in septic patients. This correlation merits further exploration.

**Supplementary Information:**

The online version contains supplementary material available at 10.1186/s13613-024-01251-z.

## Introduction

The potential effect of acetaminophen (ACT) on the endothelium has been studied in condition where, for different reasons, red blood cells are damaged and circulating cell-free hemoglobin (Hb) is detectable in plasma. In all these conditions (hemolytic and inflammatory diseases as sickle cellular disease, and invasive treatments like coronary bypass surgery or hemodialysis) high levels of extracellular hemoglobin were associated with detrimental effects on organ function and with negative outcomes [1–4].

Cell-free hemoglobin is also detected in septic plasma, where its presence represents the consequence of the alteration of red blood cells membrane that facilitates the process of hemolysis and determines unload of Hb into plasma [5]. Higher levels of plasma cell-free Hb were associated with disease severity and increased mortality both on animal models of sepsis and on humans.

The precise mechanism involved is not fully understood, but cell-free Hb is implicated in activation of the endothelium and in sepsis-induced microvascular dysfunction.

Microvascular dysfunction is a critical aspect in the pathogenesis of sepsis and septic shock and it is characterized by reduced quality and increased heterogeneity of the microcirculatory flow and lower density of the normally perfused small vessels [6–8].

Acetaminophen is one of the most widely available and commonly used drugs for both antipyretic and analgesic effects. In different contexts of release of hemoproteins, ACT was able to inhibit hemoprotein-mediated lipid peroxidation and oxidative injury. This effect could be also attributed to the ability of ACT to cross-react with nitric oxide synthase (NOS) by increasing the synthesis of NO, and with cyclooxygenase (COX), by inhibiting the synthesis of prostaglandin I2 [9–12]. However, the use of ACT as antipyrexial and the temperature control itself did not demonstrate, till now, a clear effect on the outcome of critically ill patients [13].

The group of Janz et al. demonstrated that receiving acetaminophen in a context of sepsis, with increased cell-free Hb, was associated with lower plasma concentration of oxidative injury markers and lower risk of death in hospital, independent of the other risk factors for poor outcomes [14,15, 16].

On the basis of these data, we determined to evaluate the characteristics of the sublingual microcirculation and the peripheral tissue perfusion indices pre- and post-ACT infusion in a cohort of septic and septic shock patients. We measured the plasma levels of cell-free Hb and a cluster of markers of oxidative stress and of endothelial damage.

The primary endpoint of the study was to research a difference in the Perfused Vessel Density (PVD) of the sublingual microcirculation 30 min after the end of the infusion of ACT compared with the baseline PVD before infusion.

## Methods

This is a prospective observational study conducted in the University General, Respiratory and Traumatic Intensive Care Unit (ICU) of Azienda Ospedaliero-Universitaria delle Marche (Italy). The study enrolled 50 pyrexical septic and septic shock patients receiving acetaminophen on clinical judgment for treating high body temperature.

Exclusion criteria were: age < 18 years, pregnancy, conditions preventing microvascular monitoring of the sublingual mucosa (maxillofacial trauma, serious inability to jaw, copious blood loss or secretions from the mouth), use of paracetamol in the previous 12 h, hemodialysis or evident signs of hemolysis of the blood sample.

### Clinical and demographic data

For all patients we recorded demographic parameters (age, weight, gender), cause of admission in ICU and severity scores at admission (the Simplified Acute Physiology II [SAPS] Score, the Acute Physiology and Chronic Health Disease Classification System II [APACHE] score, the Sequential Organ Failure Assessment (SOFA) score), length of stay in ICU before enrollment and SOFA score at inclusion in the study, total duration of ICU stay, ICU mortality. We also recorded the source of infection and the main biochemical, immunological and microbiological data.

We predetermined three timepoints for the trial: before the administration of acetaminophen (T0), 30 min after the end of the infusion of acetaminophen (T1) and 2 h after the end of the infusion (T2). ACT was administered, as for routine practice, in 15-to-20 min infusion.

The following variables were collected for all timepoints: body temperature, invasive arterial pressure (systolic arterial pressure—SAP, diastolic arterial pressure—DAP, mean arterial pressure—MAP, heart rate (HR), arterial and central venous blood gas parameters (in particular arterial lactate, central venous saturation—ScvO2). Vasoactive medications were also recorded.

The overall treatment of the patients conformed with the standards of Good Clinical Practice (GCP) and followed the Surviving Sepsis Campaign guidelines.

### Microvascular and NIRS-derived measurements

At T0, T1 and T2 we evaluated the sublingual microcirculation with incident dark field imaging (IDF) technology (CytoCam, Braedius Medical B.V., Huizen, the Netherlands). The video acquisition and analysis complied with the Second Consensus Conference report on the assessment of sublingual microcirculation in critically ill patients (European Society of Intensive Care Medicine) [16]. The analysis was performed offline with the Automated Vascular Analysis software (AVA v3.2, Microvision Medical, Amsterdam, NL) by an experienced operator fully blind to the intervention.

All the microcirculatory parameters were evaluated for small (diameter < 20 μm) and total (diameter < 100 μm) vessels. Perfused vessel density (PVD—primary endpoint of this study) is considered an index of functional vessel density and is calculated as the total length of perfused vessels divided by the total surface of the analyzed area. Proportion of Perfused Vessel (PPV) is a score of perfusion quality and is evaluated as the number of perfused vessels divided by total length of vessels in percent; Total Vessel Density and De Backer score are indices of vessel density (semi-quantitative essays); MFI (Microvascular Flow Index) is a measure of perfusion quality that is provided by dividing the images into four quadrants, analyzing the predominant type of flow (continuous, sluggish, intermittent, no flow) inside each quadrant and determining the average flow. Flow Heterogeneity Index (FHI) is a parameter of perfusion heterogeneity and is calculated as the difference between the highest and the lowest MFI, divided by the mean MFI [17].

At the same timepoints (T0, T1 and T2), the peripheral oxygen saturation (StO2) was measured with a 15-mm-spaced probe applied at thenar eminence though near infrared spectroscopy (NIRS- InSpectra StO2 Tissue Oxygenation Monitor, model 650; Hutchinson Technology, Hutchinson, MN, USA). To explore tissue response to a standardized ischemic insult, a vascular occlusion test (VOT) was performed through the inflation of a sphygmomanometer cuff 50 mm Hg above the systolic arterial pressure, and kept inflated until the desaturation curve reached a value of StO2 of 40%. The StO2 downslope (%/min) is calculated from the regression line of the StO2 decay during vascular occlusion and provides an index of O2 extraction (consumption rate). The StO2 upslope (%/min) is calculated from the regression line of the reperfusion phase and reflects microvascular reactivity, capillary recruitment and post-ischemic vasodilatation. The area under the curve (AUC) of StO2 represents the hyperemic response to the ischemic insult [18].

NIRS-derived parameters were calculated using a dedicated software (version 3.03 InSpectra Analysis Program; Hutchinson Technology Inc.)

### Measurements of cell-free Hb, of oxidative stress markers and markers of endothelial damage

At T0 and at T2, blood samples were collected from central venous or arterial access and centrifuged to obtain plasma and serum samples that were stored at – 80 ℃ for subsequent analysis. 8-Epi-prostaglandin F2-alpha was analyzed as marker of oxidative damage; syndecan-1 and glypican-3 as markers of damage of glycocalyx; endothelin-1 as marker of endothelial damage.

Cell-free Hb and nitric oxide were quantified in plasma through colorimetric assay (Drabkin’s reagent—Sigma-Aldrich, Saint Louis, Missouri, USA; Nitric Oxide Assay kit—Elabscience, Houston, Texas, USA).

Concentrations of syndecan-1 (Human SDC1 immunosorbent assay [ELISA]), glypican-3 (Human GPC3 ELISA kit), 8-epi-prostaglandin F2-alpha (8-epi-PGF2α ELISA kit) were measured in serum and endothelin-1 (Human ET-1 ELISA Kit, Elabscience, Houston, Texas, USA) was determined in plasma, in both cases by using the corresponding enzyme-linked immunoadsorbent essay (ELISA) kits (Elabscience, Houston, Texas, USA) in accordance with the instructions of the manufacturer.

### Sample size calculation

Sample size calculation was computed on the primary objective of the study: a total of 44 patients was shown to be adequate to research a difference of PVD of 2 mm/mm2 (standard deviation 2.17 pre and 4.17 post) or more, 30 min after the end of the infusion of acetaminophen compared to baseline value of PVD (T1 versus T0), with a power of 80% and an alpha error of 0.05. 50 patients were included to account for potential losses during the study.

### Statistical analysis

Statistical analysis was performed by using IBM SPSS statistic software (Version 17.0). Differences were considered significant at *p* values < 0.05.

To determine the distribution of the main variables included in the analysis, the Kolmogorov–Smirnov test was performed; accordingly, data are presented as median and interquartile ranges [IQR] or mean and standard deviation (SD) for continuous variables, number and percentage for discrete variables. Non-parametric statistics predominated in the analysis due to the non-gaussian nature of the main parameters. The Friedman test for repeated measures with Dunn’s post hoc test was used to compare repeated measures of the same variable at T0–T1–T2.

The non-parametric Mann–Whitney U test was used for comparison between independent samples.

The Spearman’s rank correlation coefficient was used to summarize the strength and direction (negative or positive) of a relationship between continuous variables.

### Ethical

The study protocol was approved by the Local Ethics Committee (Comitato Etico Regione Marche—CERM) and deposited as NCT02750163; it conformed to the principles of Helsinki declaration (last revision, Edinburgh 2000). In compliance with national applicable laws, informed consent was obtained from the subject before inclusion by signing the appropriate informed consent paperwork. In case of temporary conditions preventing patients from giving informed consent at inclusion, the patient was included in the trial after an informative step for the next of kin (given the observation nature of the study itself) and a deferred consent form was scheduled at recovery.

## Results

We enrolled 50 consecutive adult septic or septic shock patients between the end of June 2017 and June 2019; most of the patients were males (80%), with a mean age of 58 (± 16) years.

Median SOFA score at the entrance in ICU was 10 [7–11], with an APACHE II score of 16 [11; 23] and a median SAPS II score of 47 [36; 50]. Near half of the patients were major traumas (46%), 16% were admitted in ICU for sepsis, 14% for acute respiratory failure, 14% for neurological/neurosurgical diseases. At enrollment, median SOFA score was 9 [8; 11]. Of the 50 patients, 40 (80%) were septic and 10 (20%) were in septic shock at the time of enrollment.

The main focus of sepsis was the lower respiratory tract (56% of the patients); in 20% of the patients the genitourinary tract was the cause of sepsis. The prevalence of multiple drug resistance (MDR) was relatively low (10 patients), with a 16% of polymicrobial infections; in 7 patients the microbiology was negative (of them, 2 sepsis derived from soft tissue infections and 2 from abdominal infections).

The length of stay (LoS) in ICU was 19 [9; 26] days, with an ICU mortality of 16%, and a median LoS of non-survivors of 12 [6; 22] days.

Of the 50 patients, 9 were enrolled in the first 24 h of ICU stay. The median LOS from admission to inclusion to the study of 5 [3; 9] days.

Cell-free Hb was detectable in all patients at baseline (T0), with a median value of 1.74 [1.22; 2.94] mg/ml (min 0.17 mg/ml, max 6.82 mg/ml).

### Temperature at enrollment and plasmatic levels of acetaminophen

At enrollment, all patients had temperature ≥ 38 ℃ (38.3 ℃ [38.1; 38.8]); white blood cells count was 11 [8; 15] × 10^3^/mmc (7.8 [5.6; 10.9] × 10^3^ neutrophils/mmc, and 1.3 [0.7; 1.9] × 10^3^ lymphocytes/mmc). After the infusion of ACT, temperature significantly decreased (*p* < 0.001), the reduction was more pronounced at T2 (respective delta temperature − 0.15 ℃ [− 0.4; − 0.1] at T1 versus baseline, *p* = 0.016; − 0.5 ℃ [− 0.8; − 0.1] at T2 versus baseline, *p* > 0.001; Friedman test for repeated measures, Dunn’s post hoc test).

All the patients received ACT more than 12 h after the previous dose (if previously administered); the dose received was 11.1 [10; 12.7] mg/kg. Median plasmatic value of ACT pre-infusion was 1.04 μg/ml [0; 2.02]. Thirty minutes after the end of the infusion and after 2 h the plasmatic values were, respectively, 11.8 [9.3; 16.2] μg/ml and 7.5 [5.1; 8.4] μg/ml.

### Sublingual microvascular parameters

Of the 50 patients included in the study, 1 patient was excluded from the analysis of the sublingual microcirculation because of the low quality of images obtained (the Massey MJ et al. “Quality Index on image acquisition” was performed for all videos; Additional file 1 showing two examples of video capture and semiautomated analysis with AVA 2.0) [19].

**Fig. 1:**
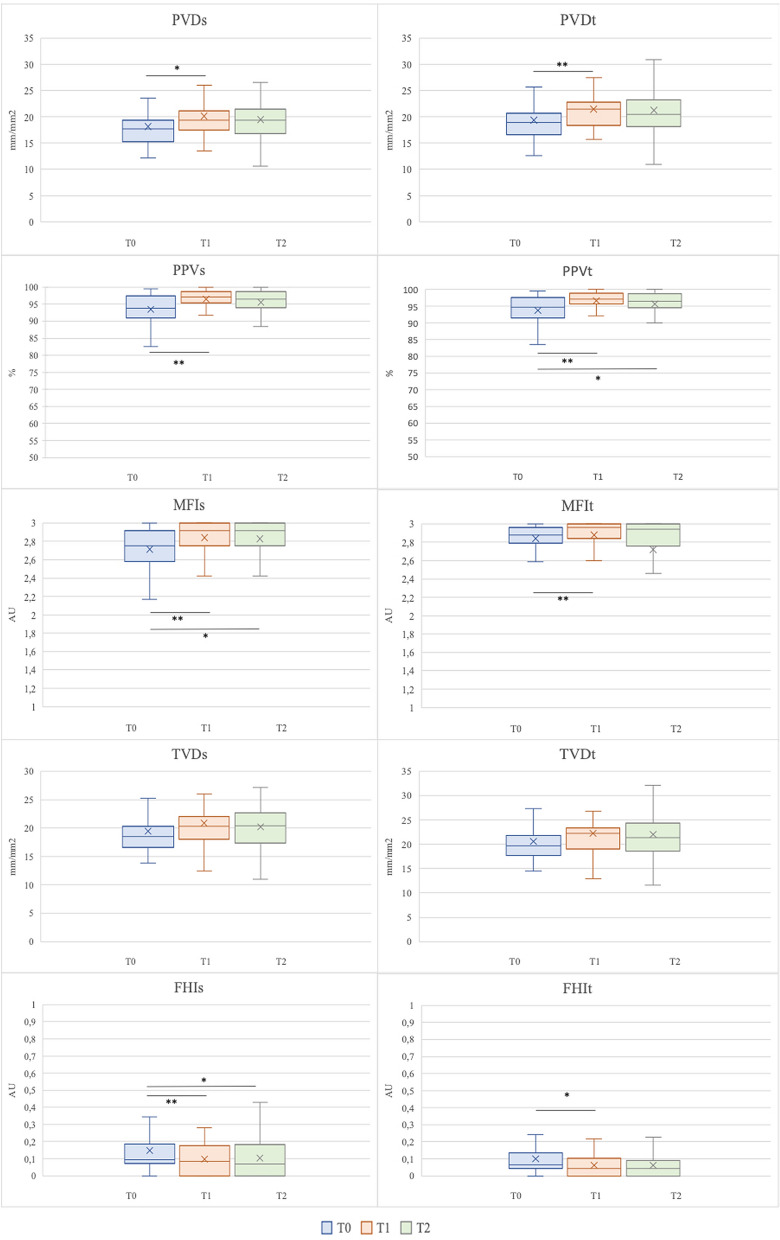
Sublingual microcirculation at T0, T1 and T2. Friedman test for repeated measures. Dunn’s post hoc test. *p < 0.05; **p < 0.01. *PVDs* perfused vessel density of small vessels, *PVDt* perfused vessel density of total vessels, *TVDs* total vessel density of small vessels, *TVDt* total vessel density of total vessels, *PPVs* Proportion of Perfused Vessels of small vessels, *PPVt* proportion of perfused vessels of total vessels, *MFIs* Microvascular Flow Index of small vessels, *MFIt* Microvascular Flow Index of total vessels, *FHIs* Flow Heterogeneity Index of small vessels, *FHIt* Flow Heterogeneity Index of total vessels

Figure 1 reports the microvascular variables at baseline (T0), 30 min after the end of the infusion of ACT (T1) and after 2 h (T2).

The Friedman’s test for repeated measures showed a statistically significant difference within-times of both the perfused vessel density of small (PVDs) and total (PVDt) vessels (respectively, *p* = 0.026 and *p* = 0.009), with an increase of both parameters at T1 versus T0 at Dunn’s post hoc test (respectively, *p* = 0.032 and *p* = 0.007). A statistically significant difference in the within-times comparison was showed for the PPV of both small and total vessels (PPVs, PPVt) and the MFIs and MFIt. The Flow Heterogeneity Index (FHI) of small and total vessels significantly decreased (respectively, *p* = 0.04 for FHIs and *p* = 0.037 for FHIt; with a significant decrease of both indices at T1 versus T0, and of FHIs also at T2 compared to baseline.

Total vessel density of small and total vessels (TVDs, TVDt), De Backer score showed a trend towards an increase that was not statistically significant at the Friedman test for repeated measures (Fig. 1, Additional  file 2).

### NIRS-derived parameters

No significant difference in StO2 was observed within-times. Similarly, the percentage of downslope StO2 after the vascular occlusion and of AUC StO2 at the deflation of sphygmomanometer cuff did not differ between T0, T1 and T2.

**Fig. 2:**
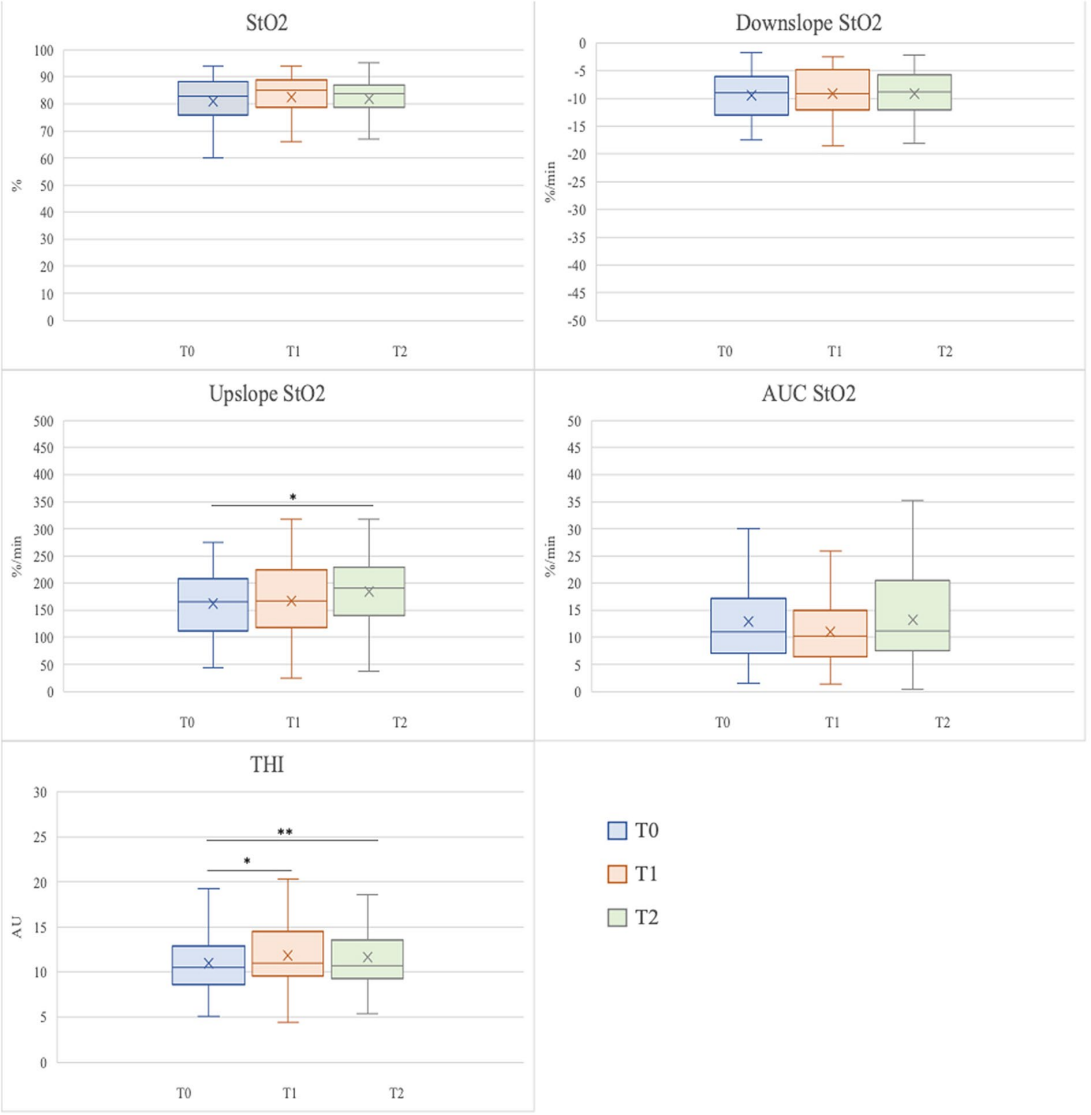
Near-infrared spectroscopy-derived variables and VOT test at T0, T1 and T2. Friedman test for repeated measures. Dunn’s post hoc test. *p < 0.05; **p < 0.01. *StO2* peripheral oxygen saturation, *AUC *area under the curve, *THI* Tissue Hemoglobin Index

The upslope StO2 showed an increase over time with a *p* = 0.047 at the Friedman test for repeated measures and a Dunn’s post hoc test significant in the comparison between T2 and T0 (*p* = 0.043). The Tissue Hemoglobin Index (THI) also increased at T1 and T2 compared to the baseline value (Fig. 2).

### Hemodynamic variables

At the Friedman test for repeated measures there was a significant decrease of systolic arterial pressure (SAP; *p* = 0.024) in particular at T2 compared to T1 (*p* = 0.049), a decrease of main arterial pressure (MAP; *p* = 0.022) and of diastolic arterial pressure (DAP; *p* = 0.005) between T1 and baseline (*p* = 0.021 for MAP, *p* = 0.005 for DAP; T1 versus T0). The heart rate (HR) decreased significantly both at T1 (*p* = 0.001) and at T2 (*p* = 0.001) compared to baseline (Friedman test for RM, *p* < 0.001). There was no need for adjunctive fluid therapy during the study period, no significant difference was evident in the comparison among times for arterial lactate, v-aPCO2 gap (veno-arterial difference in the partial pressure of carbon dioxide) and ScvO2 (venous oxygen saturation) and of need of vasoactive drugs (Table 1).

**Table 1:** Hemodynamic variables at T0, T1 and T2

	T0	T1	T2	*p*	Dunn's post hoc test
SAP, mmHg	135 [110; 139]	129 [99; 144]	130 [101; 145]	0.024	0.049 [T2-T1]
MAP, mmHg	91 [79; 94]	83 [75; 90]	84 [70; 93]	0.022	0.021 [T1-T0]
DAP, mmHg	68 [64; 71]	64 [58; 67]	63 [53; 70]	0.005	0.005 [T1-T0]
HR, bpm	96 [82; 106]	79 [71; 100]	83 [71; 95]	< 0.001	0.001 [T1-T0] 0.001 [T2-T0]
Lactate, mmol/l	1.25 [1.07; 2.25]	1.20 [1; 2.25]	1.20 [1.10; 2.43]	0,605	
Pv-aCO2 gap	5 [3; 8]	5 [3, 7]	5 [2;7]	0.936	
ScvO2, %	75 [68; 80]	75 [73; 82]	75 [72; 81]	0.309	
Norepinephrine, mcg/kg/min	0.18 [0.07; 0.40]	0.18 [0.07; 0.40]	0.15 [0; 0.34]	0.323	

Friedman test for repeated measures. Dunn’s post hoc test. *p* < 0.05. *SAP* systolic arterial pressure, *MAP* mean arterial pressure, *DAP* diastolic arterial pressure, *HR* heart rate, *Pv-aCO2 gap* veno-arterial gap of CO2 pressure, *ScvO2* central venous oxygen saturation

A continuous measurement of Cardiac Index (CI) was available only in a minority of the patients (13 over 50 patients), with a median CI of 4.1 [2.7; 4.9] L/min/m^2^ at t0, 4 [2.8; 4.3] L/min/m^2^ at t1, 3.8 [2.9; 4.1] L/min/m^2^ at t2.

### Cell-free hemoglobin, nitric oxide and markers of oxidative and endothelial damage

Baseline and T2 values of cell-free Hb, of NO and of the markers of oxidative stress and endothelial damage analyzed are shown in Table 2.

**Table 2:** Cell-free hemoglobin, nitric oxide and markers of oxidative and endothelial damage at T0 and T2

	T0	T2	*p* value (Mann–Whitney U test)
Cell-free Hb, mg/ml	1.74 [1.22; 2.94]	2.02 [1.44; 2.95]	0.900
Nitric oxide, μmol/L	52.16 [24.94; 129.34]	63.98 [22.19; 139.78]	0.856
Endothelin-, pg/ml	19.86 [5.07; 35.60]	21.00 [4.23; 34.96]	0.759
Syndecan-1, ng/ml	1.49 [0.22; 4.14]	1.57 [0.25; 3.87]	0.658
Glypican-3, ng/ml	1.40 [0.29; 4.02]	1.15 [0.37; 3.77]	0.831
8-Epi-prostaglandin F2-alpha, pg/ml	483.88 [305.24; 854.76]	435.38 [338.28; 700.27]	0.282

Wilcoxon matched-pair signed-rank (2 samples) test. *p* < 0.05

For nitric oxide, 6 on 50 patients were excluded from the analysis for technical difficulties in phase of analysis.

The analysis did not evidence significant difference between T2 and T0 for none of the markers included in the study (Table 2). No correlation was shown between SOFA score and the markers explored, except for a weak direct correlation with the plasmatic level of cell-free Hb at T2 (Spearman’s correlation coefficient 0.306, *p* = 0.032).

### Subgroup analysis for comparison between sepsis and septic shock

Additional file 3 and additional figure 1 provide supplemental data on the confrontation between patients with sepsis and patients with septic shock.

## Discussion

In this study, we observed the parameters of microvascular perfusion at both sublingual and peripheral levels in 50 adult septic and septic shock pyrexical patients before and after the intravenous infusion of 1 g of acetaminophen.

The study showed a significant increase of the density of the normally perfused small and total vessels of the sublingual microcirculation 30 min after the infusion of ACT; it also showed an increase of the MFI, a decrease in the heterogeneity of the flow and a non-statistically significant trend towards an increment of the total density of the microcirculation.

At a peripheral muscular level, while the basal oxygen saturation was not changed after the infusion of ACT and the curve of desaturation during VOT was not modified, the study showed an acceleration in the reperfusion curve, after the release of the ischemic insult caused by the VOT, 30 min after the infusion of ACT, expression of a higher reactivity of the microvasculature.

The study was not able to correlate these microvascular changes to a specific pattern of markers of endothelial damage and oxidative stress, while it demonstrated a great variability in the plasmatic expression of those markers.

Acetaminophen infusion determined modest hemodynamic impact, that did not require significant vasopressor’s increase and fluid administration and did not determine variation on lactate expression, v-aPCO2 gap or ScvO2.

In the exploratory comparison between septic and septic shock patients that will be presented in Additional file 2, it appeared a divergent trend of the microcirculation after the infusion of ACT in the two groups of patients; however, the results need further confirmation.

The results presented suggest a possible association between ACT administration and the improvement of sepsis-induced microvascular dysfunction, in particular in the context of sepsis without shock.

A cause–effect relationship could not be explored, because of the observational design of this study, and for the important limitations that will be further discussed below, but some hypothesis on the role of acetaminophen on endothelial function could be generated.

First of all, as ACT affects macrohemodynamics through its vasodilatory effect, part of the variation in the parameters of microcirculation and tissue perfusion that we showed could be attributed to the hemodynamic impact of ACT. Moreover, part of the effect of ACT could be attributed to temperature drop, even if the response of pyrexia to ACT was extremely variable in our sample.

Several studies evaluated the effects of ACT on cardiac output and stroke volume in different contexts of use, and it was demonstrated that ACT can induce hypotension in critically ill patients by a reduction of both cardiac output and of systemic vascular resistance [20]. That effect was not directly correlated to the antipyretic effect of the drug, but could help to explain some of the variations of the microvascular parameters that we showed.

On the other hand, ACT also demonstrated to increase cardiac output and mean arterial pressure in animal model of ischemia and reperfusion of the myocardium [21]. These findings are contradictory and merit further interest. Unfortunately one of the important limitations of our study is that most of the patients did not receive a proper cardiac output monitoring during the study period, and ScvO2 and v-aPCO2 gap (as surrogates of the macrohemodynamic equilibrium) did not help to study the effect of ACT and to correlate it with microhemodynamic variable (Additional files 2, 3).

The role of macrohemodynamics on the homeostasis of microhemodynamics has been already studied, in particular in the resuscitation phase of different types of shock when, if the hemodynamic coherence is preserved, the normalization of systemic variables should correspond to a parallel improvement in the microvascular function and in tissue perfusion.

In sepsis however, the hemodynamic coherence is often lost, and the achievement of the resuscitation targets could not be a synonymous of reversal of shock, because the microcirculation and the organs can remain hypoperfused and unrecognized if not monitored at a microvascular level [22].

Even if this is just hypothesis generating, we believe that the impact of ACT on temperature and macrohemodynamics are not sufficient to explain the variations of the microcirculation. If the vasodilation was the only mechanisms to impact on microcirculation, Total Vessel Density (TVD) and the Backer score, that are semi-quantitative variables related to density of capillaries, should have been more significantly interested. On the contrary, we found the major expression in the difference of quality of the perfusion and heterogeneity of the microcirculation (PVD/PPV, MFI, FHI) and we believe therefore that a mix of effects on distribution and on convection should be considered to try to interpret the results found.

In fact, sepsis is associated with important deregulation in the hemostasis of the microcirculation that derives from multifactorial insults (nitrosative and oxidative stress, hemorheological alterations, imbalance between the levels of vasodilating and vasoconstricting substances) and that causes endothelial dysfunction and glycocalyx degradation.

The accumulation of cell-free Hb released in plasma contributes to exacerbation of tissue through more than one mechanism: first of all, through the release of the heme group that is source of oxidizable iron, a substrate of redox reactions,of nitrate’s formation and lipids peroxidation; cell-free Hb determines NO depletion and it promotes vasoconstriction and cytotoxicity, it exacerbates the inflammatory response, and induces damage of the vascular endothelium. It can be supposed, therefore, that cell-free Hb-related injury could induce adjunctive microvascular damage in the context of sepsis and septic shock, leading to cellular distress and to organ injury [4, 5, 10]. This correlation merits further research.

These effects of cell-free Hb have been already demonstrated in lung, blood–brain barrier, derma, arterial microvascular endothelial cells; it was also extensively described as mechanism of heme-mediated acute renal injury [10, 23–26].

In a previous study, we showed that adult septic patients undergoing fresh or old (< 10 or > 15 days storage, respectively) red blood transfusion developed higher levels of cell-free hemoglobin after receiving units of old red blood cells, and that increasing plasma cell-free Hb after transfusion was associated with decreasing sublingual microcirculatory density [27].

Acetaminophen is a potent inhibitor of the hemoprotein-mediated lipid peroxidation, owing the ability to reduce the ferryl-protoporphyrin radical generated with the release of hemoprotein into circulation; it is also able to cross-react with nitric oxide synthase (NOS) by increasing the synthesis of NO, and with cyclooxygenase (COX), by inhibiting the synthesis of prostaglandin I2 [10–12].

At therapeutic dose, it may counterbalance the pro-inflammatory and deleterious effects of cell-free Hb, and therefore it may protect the microcirculation, improving its homogeneity and quality of the flow, through reduction of exposure to oxidative stress.

Independently from cell-free Hb levels, a potential role of the vasculature as a target of the anti-inflammatory effects of ACT has been evaluated in the cerebral microcirculation, where it was demonstrated that ACT protects cultured brain endothelial cells from oxidative stress and oxidative-induced cell death and regulates/reduces cytokine and chemokines secretion from cultured brain [28–31].

“Resuscitating” the microcirculation is a target of therapy for those patients, but unfortunately few options are currently available to normalize microvascular flow [31]. Given the high heterogeneity of sepsis a personalized approach is needed and based on the results of this study we encourage further investigation about the effects of ACT on the microcirculation and tissue perfusion of septic patients.

It should be noted, however, that existing evidence indicates that ACT does not enhance sepsis outcome [13].

The study suffers important limitations: first of all, as already mentioned, the observational nature of the study prevents from any cause–effect evaluation, and, as the study did not include a control group into the analysis, we cannot exclude that the modifications observed in the microcirculation pre and post infusion of ACT were influenced by the time passing and by unmeasured biases. The study did not select any specific timeframe between the diagnosis of sepsis and the enrollment, therefore the patients included vary in terms of phase of sepsis, phases of response of the organism to sepsis, expression of inflammatory and of immunomodulatory markers. This element can help to explain the wide variability of the markers evidenced at baseline, but also constitutes an unmodifiable bias at baselines, limits the power of the analysis and adds confounders to the analysis itself. Moreover, the lack of cardiac output monitoring prevented a better characterization of the macrohemodynamics of the patients and of the effect of ACT on cardiac output. Although a comparison between septic and septic shock patients showed an interesting trend, the unequal sample size of the two groups, the restrained numbers of septic shock patients included and the strong variability of the sample at baseline, limit excessively the validity of those results and data on the different response between patients with sepsis and patients with septic shock need to be interpreted carefully, reproduced and eventually confirmed with randomized trial to avoid overinterpretation.

Lastly, the study looked only at patients treated with ACT for pyrexia (according to the routine care protocol of the service). It could be of interest to further evaluate the use of ACT in context of sepsis without fever.

## Conclusion

The results of the study suggest a heretofore unappreciated therapeutic potential for ACT on the microcirculation of septic pyrexial patients, while the study did not show a clear connection between exposure to ACT and cell-free hemoglobin; additional mechanisms to explain the correlation between ACT, cell-free hemoglobin and microcirculation should be investigated.

### Supplementary Information

**Additional file 1. ** Examples of semiautomated analysis of the microcirculation.

**Additional file 2. ** Descriptive of microvascular variables.

**Additional file 3. ** Subgroup analysis (sepsis and septic shock).

**Additional file 4. ** Variables in the subgroups.

## Data Availability

The data are available on reasonable request to roberta.domizi@ospedaliriuniti.marche.it.
