# Supplementary material for: Potential of acetaminophen on the sublingual microcirculation and peripheral tissue perfusion of febrile septic patients: prospective observational study
Source: Ann Intensive Care. 2024 Feb 10;14:23. doi: 10.1186/s13613-024-01251-z (PMC10858855; doi:10.1186/s13613-024-01251-z)

Examples of sublingual microcirculation, video captured with IDF imaging technology, and examples of semiautomated analysis with AVA 2.0 software. *= microvascular alterations


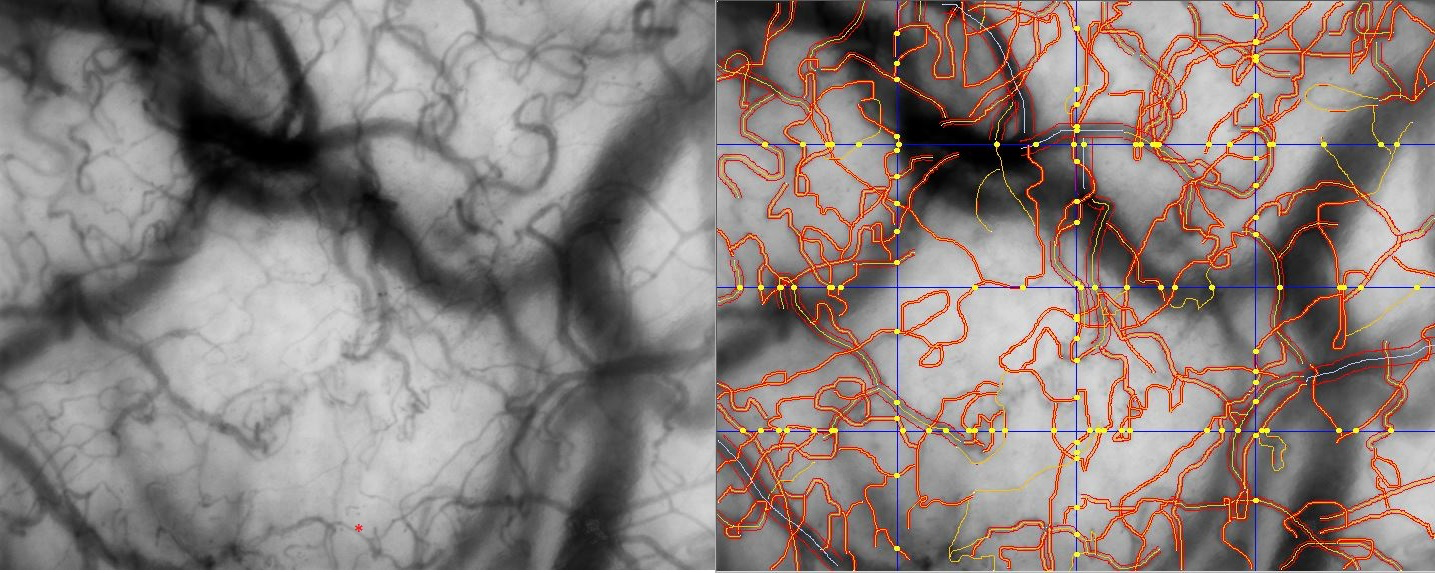


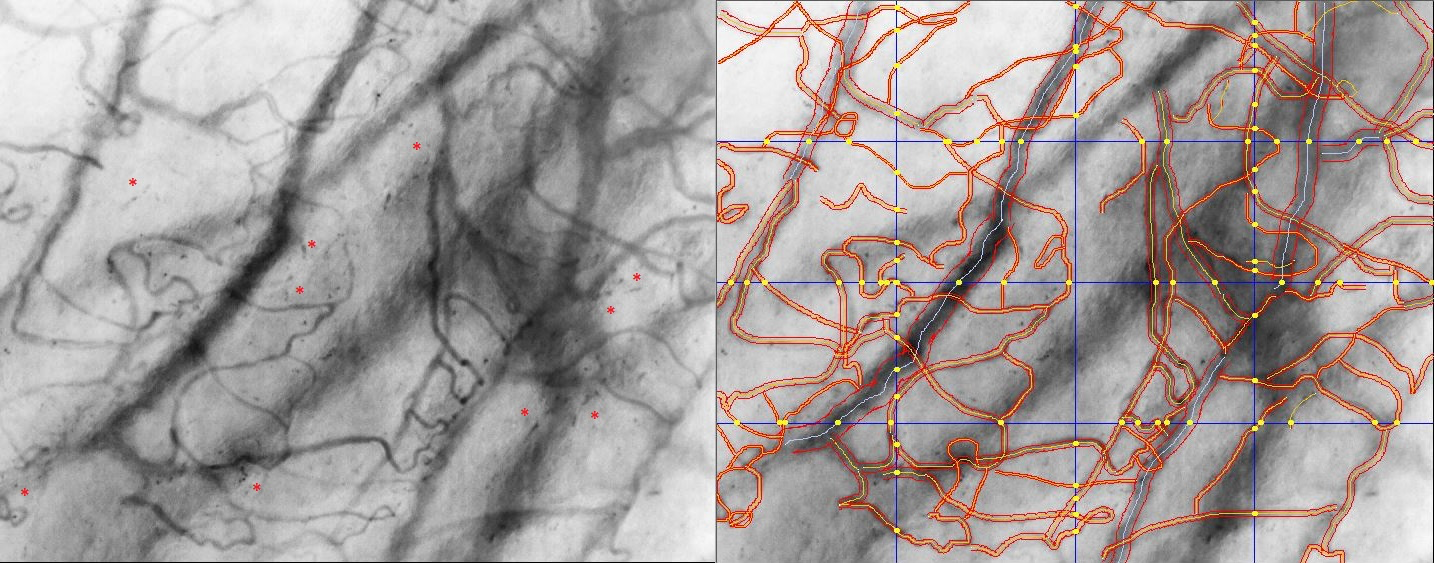

Supplement: Supplementary file 1 — Additional file 1. Examples of semiautomated analysis of the microcirculation. [file 13613_2024_1251_MOESM1_ESM.docx]
