# Supplementary material for: Potential of acetaminophen on the sublingual microcirculation and peripheral tissue perfusion of febrile septic patients: prospective observational study
Source: Ann Intensive Care. 2024 Feb 10;14:23. doi: 10.1186/s13613-024-01251-z (PMC10858855; doi:10.1186/s13613-024-01251-z)
Supplement: Supplementary file 2 — Additional file 2. Descriptive of microvascular variables. [file 13613_2024_1251_MOESM2_ESM.docx]

|  | T0 | T1 | T2 | P value | Dunn's post-hoc test |
| --- | --- | --- | --- | --- | --- |
| PVDs, mm/mm2 | 17.66  [15.05; 19.73] | 19.32  [17.20; 21.10] | 19.33  [16.78; 21.73] | *0.026* | *0.032* [T1-T0] |
| PVDt, mm/mm2 | 18.97  [16.33; 20.70] | 21.16  [18.21; 22.78] | 20.41  [17.97; 23.59] | *0.009* | *0.007* [T1-T0] |
| TVDs, mm/mm2 | 18.66  [16.56; 21.56] | 20.29  [17.56; 22.03] | 20.37  [17.19; 22.71] | 0.21 |  |
| TVDt, mm/mm2 | 19.72  [17.71; 21.95] | 21.99  [18.90; 23.45] | 21.37  [18.36; 24.47] | 0.22 |  |
| De Backer score, 1/mm | 11.43  [9.91; 13.30] | 12.12  [10.56; 13.13] | 12.11  [10.72; 13.25] | 0.15 |  |
| PPVs, % | 93.70  [90.76; 97.44] | 97.29  [95.03; 98.77] | 96.46  [93.99; 98.67] | *0.002* | *0.002* [T1-T0] |
| PPVt, % | 94.27  [90.86; 97.57] | 97.22  [95.10; 98.92] | 96.49  [94.33; 98.68] | *<0.001* | *<0.001* [T1-T0]; *0.024* [T2-T0] |
| MFIs, AU | 2.75  [2.58; 2.92] | 2.92  [2.75; 3.00] | 2.92  [2.75; 3.00] | *<0.001* | *<0.001* [T1-T0]; *0.011* [T2-T0] |
| MFIt, AU | 2.88  [2.77; 2.96] | 2.96  [2.82; 3.00] | 2.96  [2.77; 3.00] | *0.003* | *0.009* [T1-T0] |
| FHIs, AU | 0.097 [0.077; 0.191] | 0.086 [0.00; 0.177] | 0.070 [0.086; 0.188] | *0.004* | *0.005* [T1-T0];  *0.038* [T2-T0] |
| FHIt, AU | 0.086 [0.042; 0.137] | 0.042 [0.00; 0.105] | 0.042 [0.00; 0.11] | *0.037* | *0.034* [T1-T0] |

Table 1: Descriptive of the microvascular variables analyzed at T0, T1 and T2. Friedman test for Repeated Measures. Dunn’s post hoc test. p<0.05 PVDs= Perfused Vessel Density of small vessels; PVDt= Perfused Vessel Density of total vessels; TVDs= Total Vessel Density of small vessels; TVDt= Total Vessel Density of total vessels; PPVs= Proportion of Perfused Vessels of small vessels; PPVt= Proportion of Perfused Vessels of total vessels; MFIs= Microvascular Flow Index of small vessels; MFIt= Microvascolar Flow Index of total vessels; FHIs= Flow Heterogeneity Index of small vessels; FHIt= Flow Heterogeneity Index of total vessels

|  | T0 | T1 | T2 | P value | Dunn's post-hoc test |
| --- | --- | --- | --- | --- | --- |
| StO2, % | 83.00  [76.00; 89.00] | 85.00  [79.00; 89.00] | 83.80  [78.00; 87.00] | 0.208 |  |
| Downslope StO2, %/min | -9.26  [-13.17; -6.09] | -9.56  [-12.13; -4.90] | -9.08  [-12.26; -5.75] | 0.829 |  |
| Upslope StO2, %/min | 165.93  [112.50; -212.18] | 172.14  [118.36; 227.14] | 191.82  [141,27; 232,50] | *0.047* | *0.043*  [T2-T0] |
| AUC StO2, %/min | 11.60  [7.40; 18.10] | 10.30  [6.40; 15.60] | 11,20  [7.50; 20.90] | 0.500 |  |
| THI, AU | 10.40  [8.50; 12.90] | 10.70  [9.40; 14.50] | 10.60  [9.10; 13.50] | *0.003* | *0.004*  [T2-T0]  *0.028*  [T1-T0] |

Table2: Descriptive of the Near-Infrared Spectroscopy-derived variables at T0, T1 and T2. Friedman test for Repeated Measures. Dunn’s post hoc test. Significance for p<0.05. StO2= peripheral oxygen saturation; AUC=Area Under the Curve; THI= tissue hemoglobin Index
