# Supplementary material for: Potential of acetaminophen on the sublingual microcirculation and peripheral tissue perfusion of febrile septic patients: prospective observational study
Source: Ann Intensive Care. 2024 Feb 10;14:23. doi: 10.1186/s13613-024-01251-z (PMC10858855; doi:10.1186/s13613-024-01251-z)
Supplement: Supplementary file 3 — Additional file 3. Subgroup analysis (sepsis and septic shock). [file 13613_2024_1251_MOESM3_ESM.docx]

Subgroup analysis for exploratory comparison between septic and septic shock patients:

Septic and septic shock patients received comparable pro/kg doses of ACT and reached comparable plasmatic levels of the drug. No significant difference was evident in baseline temperature before ACT infusion; however, at T2 septic shock patients showed a significantly higher blood temperature compared to septic ones (38.2 [38; 39] °C versus 37.8 [37.4L; 38.4]°C, *p=0.049*, Mann-Whitney U test)*.* the delta of temperature was similar in the two groups, both at t1 and at t2 confronted with baseline temperature (delta-temperature t1-t0 -0.2°C [-0.5; 0] in septic patients and -0.15°C [-0.3;+0.4] in septic shocks; delta-temperature t2-t0 -0.6°C [-0.9; -0.1] in septic patients and -0.3°C [-0.5; - 0.05] in sept shocks).

Supplemental table 1 synthetase the median values of microvascular parameters and NIRS-derived indices in the two groups sepsis/ septic shock. The Friedman test for repeated measure with Dunn’s post hoc tests showed a significant increase of PPV and MFI of small and total vessels both at T2 and T1 compared to baseline value. This trend was non-significant in the septic shock group. FHI of small and total vessels decreased significantly in septic patients both at T1 and at T2 in comparison to baseline value.

Supplemental table 2 and 3 report data about macrohemodynamic variables and marker of oxidative stress and endothelial damage at T0, T1 and T2 in the two subgroups. Supplemental figure 1 is representative of the individual variations of PVDs (main objective of the study), temperature and macrohemodynamic variables in the patients, divided between septic (blue color) and septic shock ones (red color).

Discussion:

In this exploratory comparison that we performed dividing patients between septic and septic shock ones, it appeared a divergent trend of the variations of the microcirculation after the infusion of ACT, with an improvement of the microvascular perfusion and density in the group of septic patients, that was not evident in septic shocks. In the small sample of septic shock patients, the effect of ACT was less conclusive but tendentially detrimental on the sublingual microcirculation.

The plasmatic markers of endothelial damage and oxidative stress were not statistically different between groups, even if they showed smaller but interesting differences both at baseline and at T2, that suggest some evidence of divergent response to ACT infusion in septic patients and septic shock.

The results of the plasmatic markers of endothelial and oxidative damage are not conclusive to hypothesize a clear mechanism of ACT on the microcirculation or the glycocalix; however, we can suggest that the non-significant but evident increase of the plasmatic levels of NO in the subgroup of septic patients after ACT infusion compared to the septic shock group should be furtherly investigated to understand the role of ACT as modulator of NO production in sepsis. [1-3]

This subanalysis represents just an exploratory assessment, and the results showed have to be considered very cautiously and confirmed with further investivation, because this subanalysis is underpowered to be conclusive, and in particular the group of septic shock patients in extremely limited in number.

We also suggest to evaluate the role of the different baseline level of NO in septic shock compared to sepsis (already showed in literature) as possible explanation of the different response of the microcirculation in the two subgroups (ACT-responsive and not) other than the affect related to cell-free Hb.

NO plays a central role in maintaining the homeostasis of the microcirculation because it regulates microvascular tone and microvascular permeability and it sustains leukocyte adhesion, platelet aggregation, and endothelial integrity. During sepsis, the inflammatory response induces a systemic NO production through the up-regulation of inducible nitric oxide synthase (iNOS). In spite of the increased production, NO can be heterogeneously expressed within the same organ and tissue and can be also heterogeneously consumed by ROS, leading to a potential for relative NO deficiency.

It is demonstrated that impeding NO production in severe sepsis can potentially lead to some improvement in the macrohemodynamic response due to the block of NO-induced relaxation of vascular tone, but it also determines deleterious effect on the splanchnic and sublingual microcirculation that brings to cellular distress and to organ injury. [2-3] Therapeutic strategies based on NO donors have been studied with unconclusive results: in two clinical studies of septic patients, the sepsis-induced alteration of the sublingual microcirculation was reversed with topical administration of acetylcholine, and intravenous nitroglycerin (NO donors). [4-5] Those studies suggested that a NO-donor could be evaluated if the endothelium is NO-responsive.

On the contrary, a randomized controlled trial by EC Boerma et al found that nitroglycerin infusion (versus placebo) was not associated with improved microvascular flow in any category of vessel explored potentially for reduced nitroglycerin bioactivation (failure to convert nitroglycerin to NO in vivo) [6]

The role of ACT in contrasting NO-depletion due to cell-free Hb and in affecting the sepsis-induced microcirculatory injury merit consideration because it could represent a novel therapeutic strategy to target microvascular disfunction in sepsis.

References:

1. De Backer D, Creteur J, Preiser JC, Dubois MJ, Vincent JL. Microvascular blood flow is altered in patients with sepsis. Am J Respir Crit Care Med. 2002 Jul 1;166(1):98–104.
2. Cunha FQ, Assreuy J, Moss DW, et al. Differential induction of nitric oxide synthase in various organs of the mouse during endotoxaemia: role of TNF-alpha and IL-1-beta. Immunology. 1994 Feb;81(2):211–215.
3. Morin MJ, Unno N, Hodin RA, Fink MP. Differential expression of inducible nitric oxide synthase messenger RNA along the longitudinal and crypt-villus axes of the intestine in endotoxemic rats. Crit Care Med. 1998 Jul;26(7):1258–1264.
4. Trzeciak S, Cinel I, Phillip Dellinger R, et al. Resuscitating the microcirculation in sepsis: the central role of nitric oxide, emerging concepts for novel therapies, and challenges for clinical trials. Acad Emerg Med. 2008;15(5):399-413. doi:10.1111/j.1553-2712.2008.00109.x
5. Lambden S. Bench to bedside review: therapeutic modulation of nitric oxide in sepsis-an update. Intensive Care Med Exp. 2019;7(1):64. Published 2019 Dec 2. doi:10.1186/s40635-019-0274-x
6. Boerma EC, Koopmans M, Konijn A, Kaiferova K, Bakker AJ, van Roon EN, Buter H, Bruins N, Egbers PH, Gerritsen RT, Koetsier PM, Kingma WP, Kuiper MA, Ince C. Effects of nitroglycerin on sublingual microcirculatory blood flow in patients with severe sepsis/septic shock after a strict resuscitation protocol: a double-blind randomized placebo controlled trial. Crit Care Med. 2010;38:93–100. doi: 10.1097/CCM.0b013e3181b02fc1.

|  |  | T0 | T1 | T2 | p value |
| --- | --- | --- | --- | --- | --- |
| PVDs, mm/mm2 |  |  |  |  |  |
|  | Sepsis | 17.81 [15.23; 19.85] | 20.08 [17.96; 21.32] * | 19.75 [17.59; 22.23] * | *0.007* |
|  | Septic Shock | 17.50 [13.22; 19.70] | 16.73 [14.68; 20.49] | 15.35 [13.21; 19.97] | 0.717 |
| PVDt, mm/mm2 |  |  |  |  |  |
|  | Sepsis | 19.36 [16.62; 20.70] | 21.41 [19.26; 23.02] ** | 21.22 [18.55; 23.94] * | *0.002* |
|  | Septic Shock | 18.27 [14.32; 21.25] | 19.00 [ 16.63; 21.96] | 17.00 [ 12.80; 20.95] | 0.121 |
| TVDs, mm/mm2 |  |  |  |  |  |
|  | Sepsis | 19.11 [16.79; 21.99] | 20.41 [18.35; 22.10] | 20.96 [18.16; 23.38] | *0.021* |
|  | Septic Shock | 17.77 [14.93; 20.83] | 16.93 [14.74; 20.94] | 15.79 [12.87; 20.39] | 0.169 |
| TVDt, mm/mm2 |  |  |  |  |  |
|  | Sepsis | 19.89 [18.16; 21.99] | 22.34 [19.31; 23.91] | 22.41 [19.59; 24.76] * | 0.061 |
|  | Septic Shock | 19.58 [15.30; 22.24] | 19.00 [16.85; 22.64] | 17.45 [14.14; 21.37] | 0.236 |
| DeBacker score, 1/mm |  |  |  |  |  |
|  | Sepsis | 11.54 [10.12; 13.45] | 12.30 [11.07; 13.49] | 12.32 [11.27; 13.54] | 0.132 |
|  | Septic Shock | 10.14 [8.75; 12.23] | 10.43 [8.84; 11.83] | 10.10 [8.02; 12.22] | 0.641 |
| PPVs, % |  |  |  |  |  |
|  | Sepsis | 93.46 [90.69; 97.10] | 96.76 [96.73; 98.37] ** | 96.27 [94.05; 98.34] * | *0.003* |
|  | Septic Shock | 95.53 [91.64; 99.24] | 98.37 [97.12; 99.52] | 97.22 [91.16; 99.41] | 0.459 |
| PPVt, % |  |  |  |  |  |
|  | Sepsis | 93.73 [90.67; 97.56] | 96.93 [94.92; 98.38] ** | 96.39 [94.53; 98.29] * | *0.003* |
|  | Septic Shock | 96.24 [91.86; 98.36] | 98.08 [96.96; 99.62] | 97.41 [91.99; 99.44] | 0.169 |
| MFIs, AU |  |  |  |  |  |
|  | Sepsis | 2.75 [2.58; 2.92] | 2.92 [2.67; 3.00] ** | 2.92 [2.75; 3.00] ** | *0.001* |
|  | Septic Shock | 2.92 [2.63; 2.96] | 3.00 [2.88; 3.00] | 2.92 [2.67; 3.00] | 0.480 |
| MFIt, AU |  |  |  |  |  |
|  | Sepsis | 2.88 [2.75; 2.96] | 2.96 [2.79; 3.00] ** | 2.96 [2.75; 3.00] | *0.005* |
|  | Septic Shock | 2.85 [2.75; 2.88] | 2.96 [2.81; 3.00] | 2.96 [2.83; 3.00] | 0.485 |
| FHIs, AU |  |  |  |  |  |
|  | Sepsis | 0.097 [0.068; 0.231] | 0.086 [0.00; 0176] ** | 0.070 [0.00; 0.182] * | *0.022* |
|  | Septic Shock | 0.090 [0.064; 0.124] | 0.043 [0.00; 0.176] | 0.086 [0.00; 0.235] | 0.761 |
| FHIt, AU |  |  |  |  |  |
|  | Sepsis | 0.086 [0.042; 0.14] | 0.042 [0.032; 0.104] * | 0.034 [0.00; 0.184] * | *0.003* |
|  | Septic Shock | 0.066 [0.042; 0.09] | 0.043 [0.00; 0.103] | 0.042 [0.00; 0.099] | 0.703 |
| StO2, % |  |  |  |  |  |
|  | Sepsis | 83.50 [78.00; 88.25] | 85.00 [79.75; 89.25] | 83.90 [79.00; 87.25] | 0.192 |
|  | Septic Shock | 83.00 [64.50; 89.50] | 84.00 [68.00; 88.50] | 83.00 [73.50; 87.00] | 0.920 |
| Downslope StO2, %/min |  |  |  |  |  |
|  | Sepsis | -9.17 [-13.30; -7.73] | -10.14 [-12.28; -4.78] | -9.47 [-12.34;-5.72] | 0.924 |
|  | Septic Shock | -9.26 [-13.02; -6.08] | -7.86 [-13.16; -1.67] | -8.02[-12.05;-5.84] | 0.741 |
| Upslope StO2, %/min |  |  |  |  |  |
|  | Sepsis | 165 [112; 220] | 180 [118; 238] | 197 [141; 243] | *0.041* |
|  | Septic Shock | 180 [122; 203] | 146 [95; 193] | 176 [127; 215] | 0.670 |
| AUC StO2, %xmin |  |  |  |  |  |
|  | Sepsis | 11.70 [7.45; 18.65] | 10.45 [6.60; 15.00] | 11.25 [7.78; 20.38] | 0.320 |
|  | Septic Shock | 10.20 [4.00; 16.10] | 9.70 [5.35; 17.00] | 8.20 [5.60; 22.35] | 0.717 |
| THI, AU |  |  |  |  |  |
|  | Sepsis | 10.75 [8.97; 13.30] | 11.90 [9.70; 14.70] | 11.35 [9.33; 14.20] | *0.006* |
|  | Septic Shock | 9.30 [6.70; 12.20] | 10.30 [6.55; 11.45] | 9.80 [8.65; 11.80] | 0.273 |

Supplemental table 1: Descriptive of NIRS and microvascular indices at T0, T1 and T2 in the groups of septic (n=40) and septic shock (n=9) patients. Friedman test for Repeated Measures, with Dunn’s post hoc test. p<0.05. (* p<0.05; ** p<0.01 at Dunn’s post hoc test to T0). PVDs= Perfused Vessel Density of small vessels; PVDt= Perdused Vessel Density of total vessels; TVDs= Total Vessel Density of small vessels; TVDt= Total Vessel Density of total vessels; PPVs= Proportion of Perfused Vessels of small vessels; PPVt= Proportion of Perfused Vessels of total vessels; MFIs= Microvascular Flow Index of small vessels; MFIt= Microvascular Flow Index of total vessels; FHIs= Flow Heterogeneity Index of small vessels; FHIt= Flow Heterogeneity Index of total vessels; StO2= peripheral oxygen saturation; AUC=Area Under the Curve; THI= tissue hemoglobin Index.

|  |  | T0 | T1 | T2 | p value |
| --- | --- | --- | --- | --- | --- |
| SAP, mmHg |  |  |  |  |  |
|  | Sepsis | 133 [117; 144] | 128 [111; 138] | 128 [117; 146] ** | 0.025 |
|  | Septic Shock | 137 [120; 143] | 131 [97; 146] | 126 [103; 143] | 0.584 |
| MAP, mmHg |  |  |  |  |  |
|  | Sepsis | 85 [79; 95] | 85 [75; 89] * | 80 [75; 89] | 0.03 |
|  | Septic Shock | 89 [72; 95] | 81 [64; 90] | 81 [64; 90] | 0.202 |
| DAP, mmHg |  |  |  |  |  |
|  | Sepsis | 65 [58; 70] | 60 [55; 66] * | 61 [55; 71] | 0.031 |
|  | Septic Shock | 60 [55; 69] | 61 [50; 66] | 62 [50; 69] | 0.105 |
| HR, bpm |  |  |  |  |  |
|  | Sepsis | 88 [76; 104] | 80 [70; 95] * | 82 [69; 92] ** | 0.001 |
|  | Septic Shock | 97 [81; 113] | 96 [76; 116] | 90 [73; 106] | 0.199 |
| ScvO2, % |  |  |  |  |  |
|  | Sepsis | 80 [69; 80 ] | 76 [72; 81] | 76 [72; 78] | 0.723 |
|  | Septic Shock | 81 [ 73; 85] | 82 [75; 85] | 81 [74; 85] | 0.182 |
| vaCO2gap |  |  |  |  |  |
|  | Sepsis | 5 [3; 8] | 7 [3; 7] | 5.5 [2;7] | 0.83 |
|  | Septic Shock | 4.5 [3; 6.2] | 5 [3; 7.5] | 5 [3.5; 6.5] | 0.17 |
| Norepinephrine, mcg/kg/min |  |  |  |  |  |
|  | Sepsis | 0.11 [0; 0.29] | 0.14 [0; 0.3] | 0.11 [0; 0.29] | 0.09 |
|  | Septic Shock | 0.18 [0.09; 0.4] | 0.19 [0.09; 0.4] | 0.11 [0.05; 0.36] | 0.06 |

Supplemental table 2: Hemodynamic variables at T0, T1 and T2 in the groups of septic and septic shock patients. Friedman test for Repeated Measures, with Dunn’s post hoc test. p<0.05. SAP= Systolic Arterial Pressure; MAP= Mean Arterial Pressure; DAP= Diastolic Arterial Pressure; HR= Heart Rate; Pv-aCO2 gap= veno-arterial gap of CO2 pressure; ScvO2= central venous Oxygen Saturation. *p<0.05 T1-T0; **p<0.05 T2-T0

|  |  | T0 | T2 |
| --- | --- | --- | --- |
| Cell-free Hb, mg/ml |  |  |  |
|  | Sepsis | 1.72 [1.20-2.95] | 1.96 [1.20-2.51] |
|  | Septic Shock | 1.76 [1.36-3.06] | 2.97 [1.55-3.19] |
| NO, μmol/L |  |  |  |
|  | Sepsis | 53.84 [44.03-164.65] | 71.13 [28.78-196.81] |
|  | Septic Shock | 116.66 [46.00-175.35] | 116.03 [70.17-140.72] |
| Endothelin-1, pg/ml |  |  |  |
|  | Sepsis | 19.86 [4.99-31.28] | 19.41 [4.16-33.53] |
|  | Septic Shock | 25.57 [7.54-41.15] | 28.40 [3.96-38.94] |
| Sindecan-1, ng/ml |  |  |  |
|  | Sepsis | 1.23 [0.22-2.72] | 1.39 [0.36-2.67] |
|  | Septic Shock | 3.14 [0.18-5.88] | 4.17 [0.37-5.79] |
| Glypican-3, ng/ml |  |  |  |
|  | Sepsis | 1.30 [0.28-3.44] | 0.96 [0.33-3.32] |
|  | Septic Shock | 2.84 [0.58-5.99] | 2.79 [0.34-5.91] |
| 8-Epi-Prostaglandin F2-alpha, pg/ml |  |  |  |
|  | Sepsis | 508.41 [323.91-941.59] | 423.40 [329.55-676.12] |
|  | Septic Shock | 412.37 [249.50-631.41] | 522.55 [352.30-813.61] |

Supplemental table 3: Descriptive of the serum and plasmatic markers explored at T0, T1 and T2 in the groups of septic and septic shock patients. Friedman test for Repeated Measures, with Dunn’s post hoc test used inside the same group for comparison within-times. p<0.05


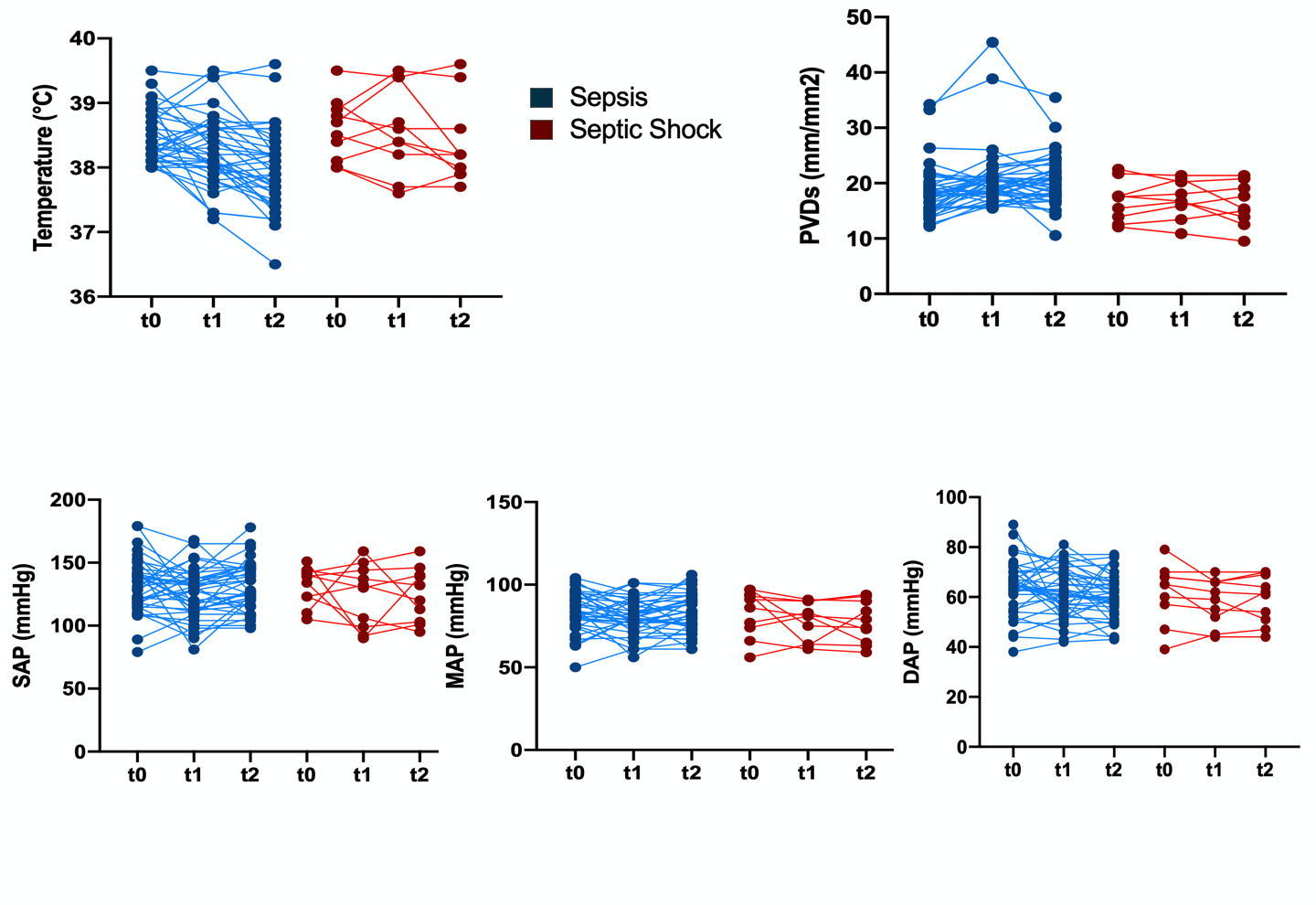


Additional figure 2: temperature, PVS of small vessels, systolic, diastolic Andean arterial pressure in the groups of septic and septic shock patients at T0, T1 and T2. PVDs= Perfused small Vessel Density; SAP = systolic Arterial Pressure; MAP= Mean Arterial Pressure; DAP= Diastolic Arterial Pressure
