# Supplementary figures and images for: Potential of acetaminophen on the sublingual microcirculation and peripheral tissue perfusion of febrile septic patients: prospective observational study
Source: Ann Intensive Care. 2024 Feb 10;14:23. doi: 10.1186/s13613-024-01251-z (PMC10858855; doi:10.1186/s13613-024-01251-z)

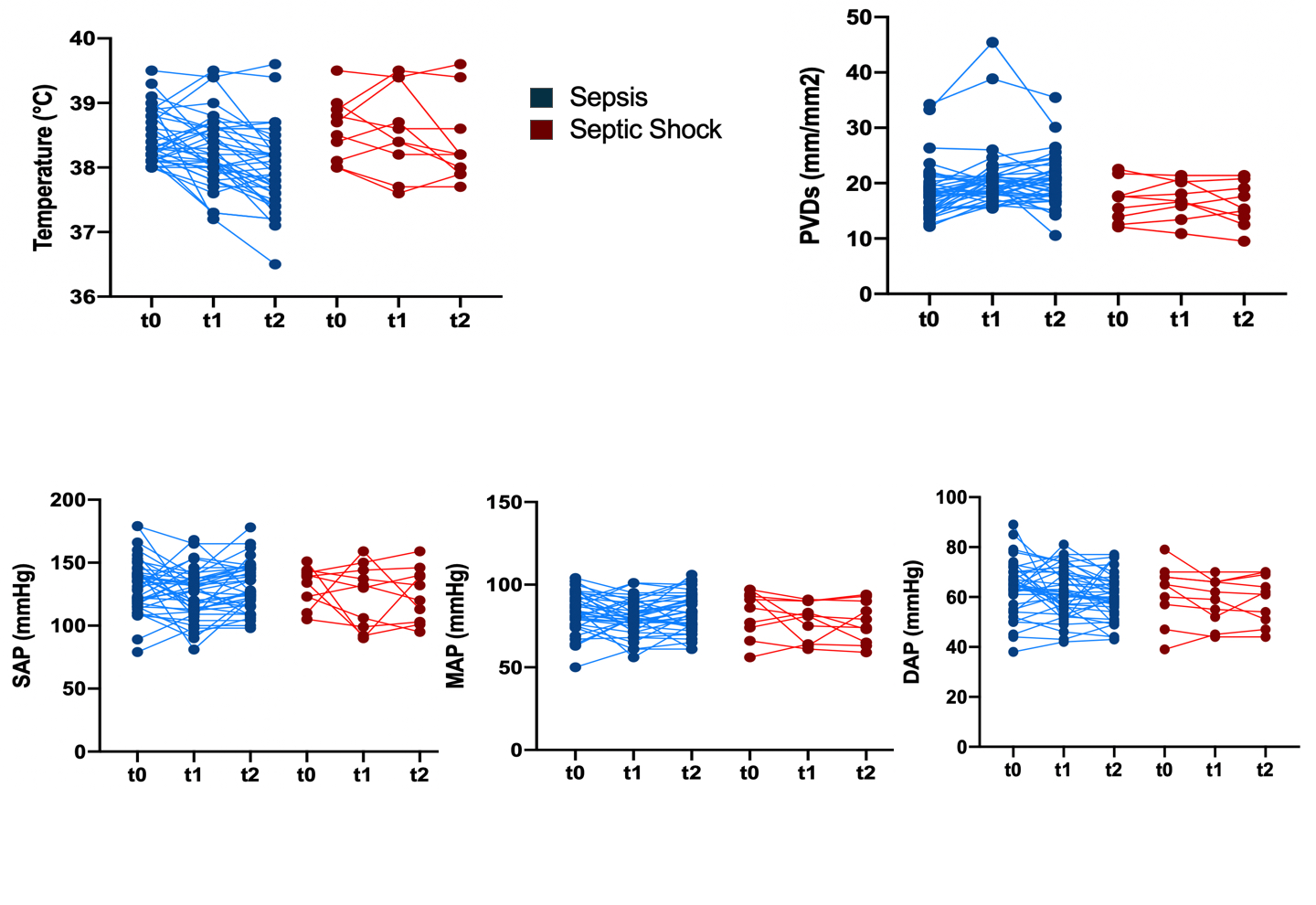

Supplement: Supplementary file 4 — Additional file 4. Variables in the subgroups. [file 13613_2024_1251_MOESM4_ESM.jpg]
